# Traumatic optic neuropathy from an air gun pellet injury: a case report.

**DOI:** 10.1093/omcr/omad130

**Published:** 2024-01-27

**Authors:** Iqra Anis, Muhammad Akbar Baig

**Affiliations:** Department of Emergency Medicine, Aga Khan University Hospital, Karachi, Pakistan; Department of Emergency Medicine, Aga Khan University Hospital, Karachi, Pakistan

## Abstract

Orbital apex fractures are a debilitating condition that can cause vision problems and are often associated with intracranial injury. Traumatic Optic Neuropathy (TON), which results in vision loss following a traumatic injury to the optic nerve, can be caused by various mechanisms, but most cases involve injuries to the globe, orbit, or adnexa. We are reporting a case of an 18 year old male with a history of an air gun being accidentally discharged into his left eye. He was evaluated and found to have an Orbital apex fracture with left eye TON. Patient was administered high dose steroids. Detailed evaluation by Ophthalmology revealed a left eye traumatic stage I macular hole which was conservatively treated with visual improvement reported during patient follow up.

## INTRODUCTION

Traumatic Optic Neuropathy (TON) is an uncommon condition resulting from acute, traumatic injury to the optic nerve. It can range from nerve contusion to complete transection, causing profound loss of vision in the affected eye. TON is usually associated with other injuries, such as orbital wall fractures or globe luxation. The incidence of optic nerve avulsion in patients with globe luxation has been reported as high as 38.2% [1]. TON can be classified as direct or indirect depending on the injury mechanism. Direct optic nerve injury involves external trauma resulting in immediate loss of vision, whereas indirect optic nerve injury involves ischemia from disruption of ophthalmic blood supply [2].

The diagnosis of TON is challenging and requires a comprehensive clinical examination. The treatment approach depends on the severity of the injury. Immediate medical attention is required in majority of cases with surgical intervention in certain cases to prevent permanent loss of vision and further damage to the optic nerve.

We would like to share our experience of a patient who suffered from optic neuropathy and vision loss due to a traumatic left orbital and maxillary fracture with preserved optic nerve.

## CASE REPORT

**Figure 1:**
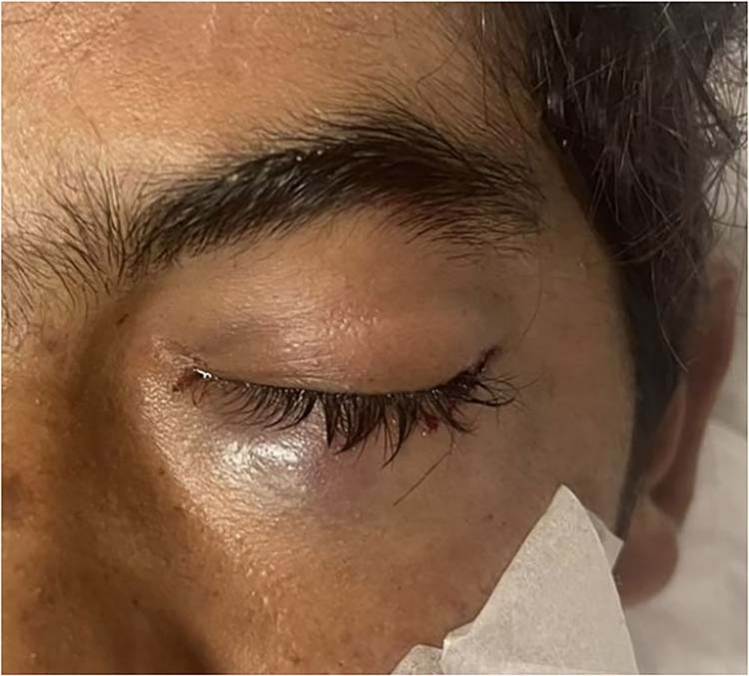
Gross external examination of left eye showing swelling and bruising affecting both upper and lower eyelids.

An 18 year old male was brought to Emergency Department with complain of left eye injury due to an accidental discharge of an air gun resulting in a penetrating pellet injury causing vision loss in left eye. On presentation to ED within 3 h of injury, he was found to be vitally stable and asymptomatic. Orbital examination revealed a swollen and bruised upper and lower eye lid with conjunctival chemosis and sub conjunctival hematoma (Fig. 1). Left eye visual acuity was limited to light perception with positive relative afferent pupillary defect (RAPD). Extraocular movements were intact. Fundoscopic examination revealed a normally appearing retina with no exudates/bleeding. Fluorescein staining of the eye did not reveal corneal and conjunctival injury or foreign bodies.

**Figure 2:**
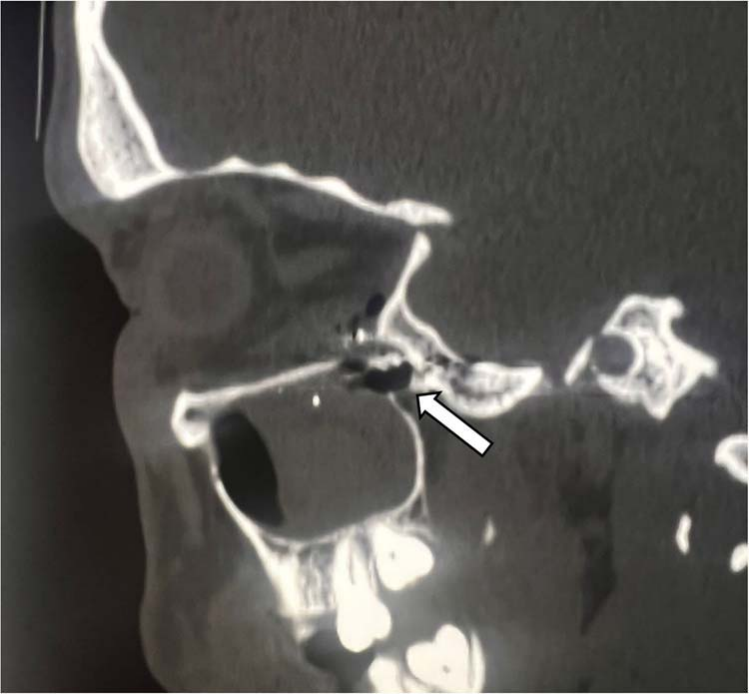
Sagittal section of CT-Head showing multiple shrapnel lodged in the left pterygopalatine fossa resulting fracture of the orbital apex (white arrow).

**Figure 3:**
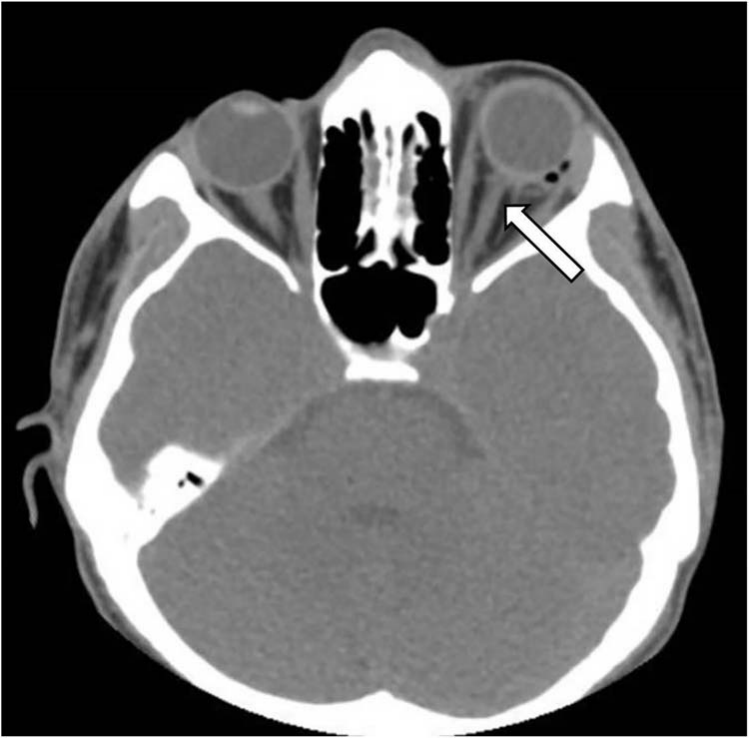
Axial section of CT-Head showing intact left optic nerve (white arrow).

Unenhanced computerized tomography (CT) scan of the brain and orbit was performed immediately which showed multiple shrapnel lodged in the left maxillary sinus, and inferior part of left orbit extending up to the inferior part of orbital apex and a large shrapnel in left pterygopalatine fossa abutting the inferolateral recess of sphenoid sinus (Fig. 2). The left globe and optic nerve was preserved and there were no signs of intracranial hemorrhage (Fig. 3). The diagnosis of direct TON was considered and the patient was given high dose steroids intravenously.

Ophthalmology team was consulted and they advised Optical Coherence Tomography that revealed a traumatic stage 1 macular hole (TMH) in left eye for which patient was conservatively treated. Weight based regimen of oral prednisolone was continued. On follow up after 2 weeks, patient reports significant improvement in his vision.

## DISCUSSION

TON is a serious complication that can result from closed head injuries, affecting 0.7%–2.5% of the population [3]. Direct TON typically leads to complete vision loss and has a poorer prognosis than indirect TON [2]. Our patient presented with Direct TON due to air gun pellet injury of the left eye leading to acute vision loss with positive RAPD.

During a comprehensive eye examination, the direct light reflex of the eye is considered to be the most reliable especially in patients presenting with impaired consciousness [4]. This reflex involves the constriction of the affected eye’s pupil when exposed to a bright light source, and an abnormal response can suggest nerve pathway damage or dysfunction resulting from structural conditions like traumatic brain injury or stroke [5].

TON can lead to a delayed diagnosis and treatment as the visual loss may not immediately become apparent and can take several months to develop after the initial trauma [6]. The management of TON is still a subject of debate among physicians, with some advocating for observation only, while others recommend high dose steroid therapy, surgical optic canal decompression, or a combination of these approaches [2]. In our case of direct TON, the patient received high dose of steroids immediately, and ophthalmology team was involved for exploring the possibility of surgical management. The speed of impact, timings between the injury and treatment, patient age group, visual acuity on presentation, presence of blood in the posterior ethmoidal cells, and loss of consciousness during injury are all factors that can affect the visual outcome of TON patients and the extent of optic nerve damage [3]. Thus, prompt diagnosis and treatment of TON are crucial in improving the chances of a positive outcome.

## References

[1] Kumari E , Chakraborty S, Ray B. Traumatic globe luxation: a case report. Indian J Ophthalmol 2015;63:682–4. 26576530 10.4103/0301-4738.169795 PMC4687199

[2] Yu-Wai-Man P, Griffiths PG. Steroids for traumatic optic neuropathy. Cochrane Database Syst Rev 2013;2013:CD006032. 10.1002/14651858.CD006032.pub2 17943877

[3] Karimi S , Arabi A, Ansari I, Shahraki T, Safi S. A systematic literature review on traumatic optic neuropathy. J Ophthalmol 2021;2021:1–10. 10.1155/2021/5553885 PMC7935564 33728056

[4] Kawasaki AK . Diagnostic approach to pupillary abnormalities. Continuum (Minneap Minn) 2014;20:1008–22. 25099106 10.1212/01.CON.0000453306.42981.94 PMC10563972

[5] Chen B , Zhang H, Zhai Q, Li H, Wang C, Wang Y. Traumatic optic neuropathy: a review of current studies. Neurosurg Rev 2022;45:1895–913. 35034261 10.1007/s10143-021-01717-9

[6] Yu-Wai-Man P . Traumatic optic neuropathy-clinical features and management issues. Taiwan J Ophthalmol 2015;5:3–8. 26052483 10.1016/j.tjo.2015.01.003 PMC4457437

